# Emergence of a multidrug-resistant and virulent *Streptococcus pneumoniae* lineage mediates serotype replacement after PCV13: an international whole-genome sequencing study

**DOI:** 10.1016/S2666-5247(22)00158-6

**Published:** 2022-10

**Authors:** Stephanie W Lo, Kate Mellor, Robert Cohen, Alba Redin Alonso, Sophie Belman, Narender Kumar, Paulina A Hawkins, Rebecca A Gladstone, Anne von Gottberg, Balaji Veeraraghavan, K L Ravikumar, Rama Kandasamy, Sir Andrew J Pollard, Samir K Saha, Godfrey Bigogo, Martin Antonio, Brenda Kwambana-Adams, Shaper Mirza, Sadia Shakoor, Imran Nisar, Jennifer E Cornick, Deborah Lehmann, Rebecca L Ford, Betuel Sigauque, Paul Turner, Jennifer Moïsi, Stephen K Obaro, Ron Dagan, Idrissa Diawara, Anna Skoczyńska, Hui Wang, Philip E Carter, Keith P Klugman, Gail Rodgers, Robert F Breiman, Lesley McGee, Stephen D Bentley, Carmen Muñoz Almagro, Emmanuelle Varon, The Global Pneumococcal Sequencing Consortium, Abdullah Brooks, Alejandra Corso, Alexander Davydov, Alison Maguire, Anmol Kiran, Benild Moiane, Bernard Beall, Chunjiang Zhao, David Aanensen, Dean Everett, Diego Faccone, Ebenezer Foster-Nyarko, Ebrima Bojang, Ekaterina Egorova, Elena Voropaeva, Eric Sampane-Donkor, Ewa Sadowy, Geetha Nagaraj, Helio Mucavele, Houria Belabbès, Naima Elmdaghri, Jennifer Verani, Jeremy Keenan, John Lees, Jyothish N Nair Thulasee Bhai, Kedibone Ndlangisa, Khalid Zerouali, Leon Bentley, Leonid Titov, Linda De Gouveia, Maaike Alaerts, Margaret Ip, Maria Cristina de Cunto Brandileone, Md Hasanuzzaman, Metka Paragi, Michele Nurse-Lucas, Mignon du Plessis, Mushal Ali, Nicholas Croucher, Nicole Wolter, Noga Givon-Lavi, Nurit Porat, Özgen Köseoglu Eser, Pak-Leung Ho, Patrick Eberechi Akpaka, Paula Gagetti, Peggy-Estelle Tientcheu, Pierra Law, Rachel Benisty, Rafal Mostowy, Roly Malaker, Samanta Cristine Grassi Almeida, Sanjay Doiphode, Shabir Madhi, Shamala Devi Sekaran, Stuart Clarke, Somporn Srifuengfung, Susan Nzenze, Tamara Kastrin, Theresa Ochoa, Waleria Hryniewicz, Yulia Urban

**Affiliations:** a Parasites and Microbes Programme, Wellcome Sanger Institute, Hinxton, UK; b ACTIV, Association Clinique et Thérapeutique Infantile du Val-de-Marne, Saint Maur-des-Fossés, France; c GPIP, Groupe de Pathologie Infectieuse Pédiatrique, Paris, France; d AFPA, Association Française de Pédiatrie Ambulatoire, Saint-Germain-en-Laye, France; e Université Paris Est, IMRB-GRC GEMINI, Créteil, France; f Clinical Research Center, Centre Hospitalier Intercommunal de Créteil, Créteil, France; g Unité Court Séjour, Petits nourrissons, Service de Néonatalogie, Centre Hospitalier Intercommunal de Créteil, Créteil, France; h National Reference Center for Pneumococci, Centre Hospitalier Intercommunal de Créteil, Créteil, France; i Department of RDI Microbiology, Institut de Recerca Sant Joan de Deu, Hospital Sant Joan de Deu, Barcelona, Spain; j School of Medicine, Universitat Internacional de Catalunya, Barcelona, Spain; k Spanish Network of Epidemiology and Public Health, CIBERESP, Instituto de Salud Carlos III, Madrid, Spain; l Rollins School Public Health, Emory University, Atlanta, GA, USA; m Emory Global Health Institute, Emory University, Atlanta, GA, USA; n Department of Biostatistics, Institute of Basic Medical Sciences, Faculty of Medicine, University of Oslo, Oslo, Norway; o Centre for Respiratory Diseases and Meningitis, National Institute for Communicable Diseases, Johannesburg, South Africa; p Department of Clinical Microbiology, Christian Medical College, Vellore, India; q Central Research Laboratory, Kempegowda Institute of Medical Sciences, Bangalore, India; r Oxford Vaccine Group, Department of Paediatrics, University of Oxford, Churchill Hospital, Oxford, UK; s NIHR Oxford Biomedical Research Centre, Oxford, UK; t School of Women and Children's Health, University of New South Wales, Sydney, NSW, Australia; u Discipline of Paediatrics and Child Health, School of Clinical Medicine, University of New South Wales, Sydney, NSW, Australia; v Child Health Research Foundation, Dhaka, Bangladesh; w Kenya Medical Research Institute, Kisumu, Kenya; x WHO Collaborating Centre for New Vaccines Surveillance, Medical Research Council Unit The Gambia at The London School of Hygiene & Tropical Medicine, Fajara, The Gambia; y NIHR Global Health Research Unit on Mucosal Pathogens, Division of Infection and Immunity, University College London, London, UK; z Microbiology and Immunology Laboratory, Department of Biology, Lahore University of Management Sciences, Lahore, Pakistan; aa Department of Pediatrics and Child Health, Aga Khan University, Karachi, Pakistan; ab Malawi-Liverpool-Wellcome-Trust, Blantyre, Malawi; ac Institute of Infection, Veterinary and Ecological Sciences, University of Liverpool, Liverpool, UK; ad Wesfarmers Centre of Vaccines and Infectious Diseases, Telethon Kids Institute, The University of Western Australia, Perth, WA, Australia; ae Papua New Guinea Institute of Medical Research, Goroka, Papua New Guinea; af Centro de Investigação em Saúde da Manhiça, Maputo, Mozambique; ag Cambodia Oxford Medical Research Unit, Angkor Hospital for Children, Siem Reap, Cambodia; ah Centre for Tropical Medicine and Global Health, Nuffield Department of Medicine, University of Oxford, Oxford, UK; ai Agence de Médecine Préventive, Abidjan, Côte d'Ivoire; aj Division of Pediatric Infectious Disease, University of Nebraska Medical Center Omaha, Omaha, NE, USA; ak International Foundation against Infectious Diseases in Nigeria, Abuja, Nigeria; al Ben-Gurion University of the Negev, Beer-Sheva, Israel; am Department of Microbiology, Faculty of Medicine and Pharmacy of Casablanca, Hassan II University of Casablanca, Casablanca, Morocco; an National Reference Laboratory, Mohammed VI University of Health Sciences, Casablanca, Morocco; ao Department of Epidemiology and Clinical Microbiology, National Medicines Institute, Warsaw, Poland; ap Peking University People ‘s Hospital, Beijing, China; aq Institute of Environmental Science and Research Limited, Kenepuru Science Centre, Porirua, New Zealand; ar Pneumonia Program, Bill & Melinda Gates Foundation, Seattle, WA, USA; as Centers for Disease Control and Prevention, Atlanta, GA, USA

## Abstract

**Background:**

Serotype 24F is one of the emerging pneumococcal serotypes after the introduction of pneumococcal conjugate vaccine (PCV). We aimed to identify lineages driving the increase of serotype 24F in France and place these findings into a global context.

**Methods:**

Whole-genome sequencing was performed on a collection of serotype 24F pneumococci from asymptomatic colonisation (n=229) and invasive disease (n=190) isolates among individuals younger than 18 years in France, from 2003 to 2018. To provide a global context, we included an additional collection of 24F isolates in the Global Pneumococcal Sequencing (GPS) project database for analysis. A Global Pneumococcal Sequence Cluster (GPSC) and a clonal complex (CC) were assigned to each genome. Phylogenetic, evolutionary, and spatiotemporal analysis were conducted using the same 24F collection and supplemented with a global collection of genomes belonging to the lineage of interest from the GPS project database (n=25 590).

**Findings:**

Serotype 24F was identified in numerous countries mainly due to the clonal spread of three lineages: GPSC10 (CC230), GPSC16 (CC156), and GPSC206 (CC7701). GPSC10 was the only multidrug-resistant lineage. GPSC10 drove the increase in 24F in France and had high invasive disease potential. The international dataset of GPSC10 (n=888) revealed that this lineage expressed 16 other serotypes, with only six included in 13-valent PCV (PCV13). All serotype 24F isolates were clustered in a single clade within the GPSC10 phylogeny and long-range transmissions were detected from Europe to other continents. Spatiotemporal analysis showed GPSC10-24F took 3–5 years to spread across France and a rapid change of serotype composition from PCV13 serotype 19A to 24F during the introduction of PCV13 was observed in neighbouring country Spain.

**Interpretation:**

Our work reveals that GPSC10 alone is a challenge for serotype-based vaccine strategy. More systematic investigation to identify lineages like GPSC10 will better inform and improve next-generation preventive strategies against pneumococcal diseases.

**Funding:**

Bill & Melinda Gates Foundation, Wellcome Sanger Institute, and the US Centers for Disease Control and Prevention.

## Introduction

Pneumococcal conjugate vaccines (PCVs) have been effective at reducing pneumococcal disease worldwide.^1^ However, increases in replacement with non-PCV serotypes remain a concern,^2^, ^3^ such as the increase of serotype 19A after introduction of PCV7 in the USA in 2000.^4^ In France, a big increase in pneumococcal meningitis cases occurred 5 years after the roll out of PCV13, mainly driven by serotype 24F.^2^, ^3^ This serotype also mediated serotype replacement in multiple other countries, such as Argentina,^5^ Canada,^6^ Denmark,^7^ Germany,^8^ Israel,^9^ Italy,^10^ Japan,^11^ Lebanon,^12^ Norway,^13^ Spain,^14^, ^15^ and the UK,^16^ and was reported to be the predominant serotype causing invasive pneumococcal disease (IPD) in Portugal^17^ after PCV13 introduction.

The serotype 24F capsule has a high invasive disease potential and propensity to cause meningitis.^18^ In France, the fatality rate for meningitis due to serotype 24F pneumococci was 13%, similar to that caused by pneumococci expressing other serotypes (11%) between 2001 and 2016.^2^ In some countries, the increase in serotype 24F was concomitant with increasing prevalence of penicillin resistance in IPD overall^2^, ^10^ and IPD due to non-vaccine serotypes.^5^ Despite serotype 24F being an important emerging serotype with high invasiveness and potential for antimicrobial resistance, it is not included in any upcoming PCV formulations (PCV15, PCV20, and PCV24).

**Research in context**

**Evidence before this study**

We searched PubMed using the terms “*Streptococcus pneumoniae*” AND “24F” OR “CC230” OR “GPSC10” for papers published in English between Jan 1, 2000, and Feb 19, 2021. We searched for population-based studies which reported changes in serotype 24F before and after the introduction of pneumococcal conjugate vaccines (PCV) in the country or region. After reviewing 59 articles, 28 met the inclusion criteria. The effects of 7-valent PCV were measured in six studies and 13-valent PCV (PCV13) in 23 studies. Most studies used samples from children or adults with invasive pneumococcal disease (IPD; n=20), four studies included isolates from both carriage and IPD cases, and four studies analysed isolates from carriage alone. Studies were conducted at the national or regional level, and isolates were typed using Quellung, latex agglutination, or PCR-based methods. Among IPD cases in children, 24F was identified as the predominant serotype post PCV13 in eight studies representing cases from France, Denmark, Spain, Italy, and Japan. For the three studies with serotype stratified by age, the predominance of 24F was only observed in children younger than 5 years. Serotype 24F was the second most common serotype, or the most common together with 12F, in four further studies of IPD cases post PCV13 in Germany, France, and the UK. An increase in 24F post PCV7 was reported among IPD cases from Spain, Italy, and France, carriage and IPD cases in Norway, and carriage in Portugal.

**Added value of this study**

We have an enhanced understanding of the multidrug resistant lineage of *Streptococcus pneumoniae* which has driven the increase in serotype 24F in France after PCV13. Additionally, we utilised a global collection of *S pneumoniae* isolates from 56 countries to contextualise the isolates from France, identifying the predominant lineage (GPSC10) associated with the increase of multidrug resistance in serotype 24F globally. This study is complemented by the knowledge we gained from previous country-specific analyses to demonstrate GPSC10 might pose a global threat after PCV13 due to a high risk of vaccine evasion.

**Implications of all the available evidence**

The increase in serotype 24F post PCV13 in France was attributed to the multidrug-resistant lineage GPSC10. Concerningly, GPSC10 has a relatively high invasive disease potential and propensity to cause meningitis, independent of serotype. GPSC10 appears to be highly capable of acquiring DNA that might result in antimicrobial resistance and serotype switches. Analyses of GPSC10 isolates from a global dataset of *S pneumoniae* genomes have identified expression of an additional 16 serotypes of which only six are included in PCV13. Antimicrobial use might have contributed to selection of GPSC10 in France and Spain, decreasing the benefit of PCV for reduction of antimicrobial resistance. GPSC10 has transmitted among European countries, with long-range transmissions to other continents. The evidence suggests that the expansion of GPSC10 might be a challenging problem to address using a serotype-based vaccine strategy.

Pneumococcal lineages driving the increase in serotype 24F varied between countries. In Argentina,^5^ Lebanon,^12^ and Spain,^19^ the increase was mainly driven by the Global Pneumococcal Sequence Cluster (GPSC) and clonal complex (CC) GPSC10 (CC230), in Denmark^7^ by GPSC6 (CC156), and in Japan by GPSC106 (CC2572).^11^ In the present study, we used whole-genome sequencing to investigate the pneumococcal lineages driving the increase in serotype 24F after PCV13 introduction in France, then placed this lineage in global context and elucidated its spatiotemporal transmission in France and across the world.

## Methods

### Study design

A representative set of serotype 24F pneumococcal isolates was randomly selected from the datasets collected through a nationwide hospital-based active surveillance for IPD^2^, ^3^ and carriage survey^20^ across France and were whole-genome sequenced. The collection included 190 isolates from invasive disease cases and 229 isolates from asymptomatic colonisation among individuals younger than 18 years, from 2003 to 2018 (appendix 1 p 6). The study period spanned the routine use of PCV7 in 2006 and PCV13 in 2010. To identify the pneumococcal lineage driving the increase in serotype 24F, we grouped isolates into GPSCs and CCs and evaluated each lineage's invasive disease potential and propensity to cause meningitis by calculating odds ratio (OR) with reference to carriage.

We then contextualised serotype 24F and the driver lineage (GPSC10) with a global collection of additional genomes from the Global Pneumococcal Sequencing (GPS) Project database (n=25 590 from 57 countries, last accessed on Oct 2, 2021). For comparison, we also sequenced a collection of 91 pneumococcal isolates that were responsible for the increase in serotype 24F in Spain. These isolates were collected from children aged younger than 5 years in the Catalan support laboratory for non-mandatory molecular surveillance of IPD located at Hospital Sant Joan de Déu, Barcelona, from 2009 to 2018.^19^ In summary, phylogenetic analysis was carried out on 642 serotype 24F isolates from 29 countries across Africa, Asia, Europe, North America, South America, and Oceania. An international collection of 888 GPSC10 isolates from 33 countries, regardless of serotype, were also included in phylogenetic, evolutionary, and spatiotemporal analyses.

### Genome sequencing and genomic characterisation

The pneumococcal isolates in this study were whole-genome sequenced using a Illumina HiSeq sequencer (Illumina, San Diego, CA, USA). The sequence reads were subjected to quality control as previously described (appendix 1 pp 4–5).^21^ We characterised each genome by assigning GPSC using PopPUNK (version 2.4.0),^21^ sequence type using MLSTcheck (version 2.1.1706216),^22^ CC using goeBURST (version 1.2.1), and serotype using SeroBA (version 1.0.0),^23^ and resistance profile against 17 antimicrobials, including penicillin, chloramphenicol, erythromycin, co-trimoxazole, and tetracycline, using a pipeline developed by the Streptococcus Laboratory at the Centers for Disease Control and Prevention, Atlanta, GA, USA.^24^ Multidrug resistance was defined as an isolate resistant to three or more antibiotic classes. In our previous large-scale analysis, we showed high concordance between GPSC and CC, therefore sequence types identified in previous studies that did not have genome data were used to infer GPSC in this study.^21^ All sequencing reads were deposited in European Nucleotide Archive (ENA) and the accession numbers and in silico output are included in appendix 2. Additional details on methodology are in appendix 1 (pp 4–5).

### Phylogenetic analysis

We performed phylogenetic analysis on all serotype 24F isolates by constructing a maximum likelihood tree using FastTree (version 2.1.10) with the general time reversible substitution model.^25^ Phylogenies were built based on single nucleotide polymorphisms (SNPs) extracted from individual alignment generated by mapping reads to a reference genome of *Streptococcus pneumoniae* ATCC 700669 (National Center for Biotechnology Information [NCBI] accession: FM211187) using SMALT (version 0.7.4), with default settings and to a reference sequence of serotype 24F capsular encoding region (ie, *cps*, NCBI accession: CR931688) using Burrows Wheeler Aligner (version 0.7.17-r1188).^26^ The phylogenetic trees were then overlaid with epidemiological data and in silico output, as described earlier, and visualised in Microreact.

### Evolutionary and spatiotemporal analysis

Sequence reads of GPSC10 (CC230) isolates were mapped to the GPSC10 reference genome Denmark^14^-32 (ENA accession: ERS1706837) using Burrows Wheeler Aligner (version 0.7.17-r1188).^26^ Recombination was detected and removed using Gubbins (version 2.4.1).^27^ A recombination-free phylogeny was produced with RAxML (version 8.2.8)^28^ and then visualised in Microreact, together with metadata. Bayesian phylogenetic analysis on a subset of serotype 24F isolates within GPSC10 was conducted to generate a time-resolved phylogeny and provide estimates of the median effective population size over time with a 95% highest posterior density to detect any exponential increase in population using BEAST Bayesian skyline model (version 2.6.3).^29^ The time-resolved phylogeny was visualised in Microreact.

To calculate the time it took for GPSC10-24F to spread across France, we inferred the evolutionary time between pairs of genomes from the time-resolved phylogeny. We used a risk ratio framework to calculate the odds that a pair of genomes from within the same province (0–50 km) compared with pairs isolated from locations between 250 and 350 km apart (the mean distance between different French provinces was 359 km with a range of 54–729 km) had a specified time-to-most-recent-common-ancestor (tMRCA) across rolling 2 year divergence time windows from 2 to 20 years.^30^ We restricted the analysis to pairs isolated within the same year to mitigate variable geographic sampling across years. A risk ratio close to 1 indicated an equal chance that a pair of isolates diverged from the same tMRCA within and between provinces. To determine uncertainty, we calculated the risk ratio using 1000 bootstrap iterations. The spatiotemporal analysis was run using R (version 3.6.0).

### Statistical analysis

We grouped the French isolates into four vaccine periods as previously described:^2^ (1) targeted PCV7 period (2003–05) in which PCV7 was only reimbursed and recommended for children in a day-care centre with two or more other children, children in families with more than two children, or children breastfed for fewer than 2 months; (2) generalised PCV7 period (2006–10) during which PCV7 was offered to all children younger than 2 years; (3) early PCV13 period (2011–14); and (4) late PCV13 period (2015–18) during which PCV7 was replaced with PCV13, without catch-up. We compared the prevalence of GPSCs between a vaccine period and the previous vaccine period, except for the earliest targeted PCV7 period. To determine if differences in proportions between groups were significant, a two-sided Fisher's exact test was used. Statistical tests were performed in R (version 3.6.0). Multiple testing correction was carried out using the Benjamini-Hochberg false discovery rate of 5%. Two-sided p values of less than 0·05 were considered significant.

### Role of funding source

The funders of the study had no role in the study design, data collection, data analysis, data interpretation, or writing of the report.

## Results

**Figure 1:**
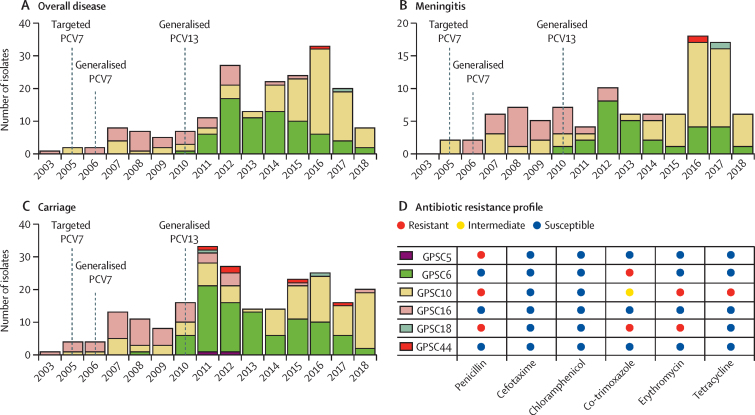
Proportion of serotype 24F pneumococcal lineages from France between 2003 and 2018 (A–C) and predominant antibiotic resistance profiles of each lineage (D) GPSC=global pneumococcal sequence cluster. PCV=pneumococcal conjugate vaccine.

The 419 French serotype 24F pneumococcal isolates were sequenced and all passed sequencing quality control. The isolates belonged mainly to three lineages: GPSC10 (CC230, 174 [41·5%]), GPSC6 (CC156, 160 [38·2%]), and GPSC16 (CC66, 74 [17·7%]). A small percentage of isolates belonged to other lineages: GPSC44 (CC177, six [1·4%]), GPSC18 (CC15, three [0·7%]), and GPSC5 (CC172, two [0·5%]). Over the study period (2003–18), clonal replacement was observed in overall disease, meningitis, and carriage isolates (figure 1). Overall, the proportion of serotype 24F isolates belonging to GPSC16 decreased significantly from 60·5% in the generalised PCV7 period (2006–10) to 10·6% in the early PCV13 period (2011–14; p<0·0001). By contrast, GPSC6 significantly increased from 9·9% in the generalised PCV7 period to 62·7% in the early PCV13 period (2011–14; p<0·0001). The proportion of 24F isolates that were GPSC6 decreased from 62·7% in the early PCV13 period to 30·2% in the late PCV13 period (2015–18; p<0·0001), and the proportion that belonged to GPSC10 significantly increased from 23·0% in the early PCV13 period to 65·1% in the late PCV13 period (p<0·0001; appendix 1 p 14). In the late PCV13 period, GPSC10 became the predominant lineage among 24F isolates, accounting for 100% of the ten pneumonia cases, 35 (74%) of 47 meningitis cases, four (67%) of six other infections, 11 (50%) of 22 bacteraemias, and 50 (60%) of 84 asymptomatic colonisation. In contrast to the pan-susceptible GPSC16, the replacement lineage GPSC6 was co-trimoxazole-resistant and GPSC10 was multidrug resistant. Stratified by IPD and carriage, we did not observe any significant difference in GPSC, sequence type, and antimicrobial resistance between children younger than 5 years and individuals aged 5–17 years (appendix 1 pp 15–17).

**Figure 2:**
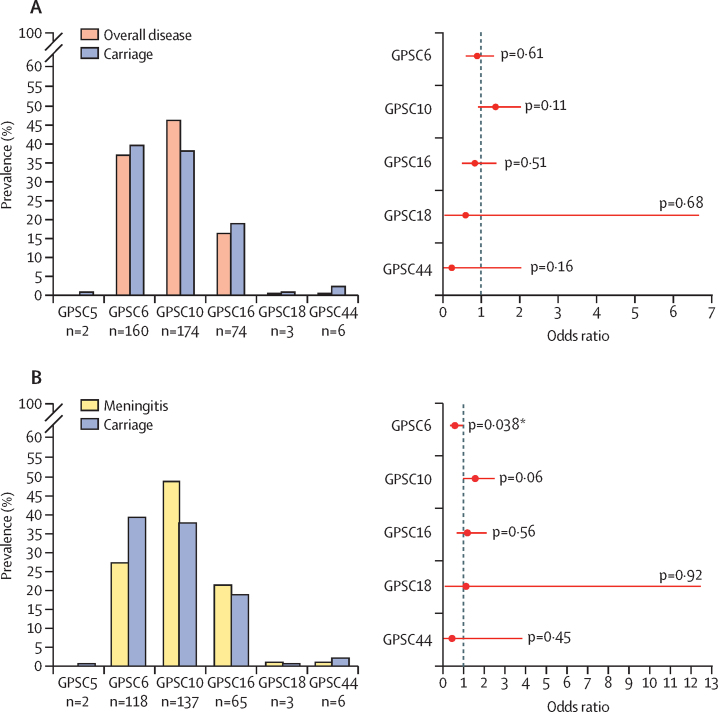
Prevalence of pneumococcal lineages by clinical manifestations and odds ratio for causing overall invasive diseases (A) and meningitis by reference to carriage (B) The odds ratio and 95% CI were calculated using Fisher's Exact test. *Indicates statistical significance. GPSC=global pneumococcal sequence cluster.

Compared with other serotype 24F lineages, GPSC10 was more frequently detected in overall invasive disease (OR 1·38 [95% CI 0·93–2·04], p=0·11) and meningitis cases (OR 1·57 [95% CI 0·98–2·51], p=0·060) than in asymptomatic colonisation. Although the finding did not reach statistical significance, it suggested that this lineage had relatively high invasive disease potential and propensity to cause meningitis (figure 2; appendix 1 p 18). By contrast, GPSC6 was more frequently identified in carriage than meningitis isolates (OR 0·58 [95% CI 0·35–0·97], p=0·038).

Analyses on an international collection of 642 serotype 24F isolates revealed that this serotype was widely distributed across 29 countries (appendix 1 p 7). These isolates were assigned into 20 GPSCs. GPSC10, GPSC16, GPSC150, and GPSC206 were the most common lineages, accounting for 439 (68%) of 642 of the overall GPS collection and 96 (78%) of 123 of a subcollection that included isolates randomly selected from disease surveillance systems and carriage surveys from 21 countries (appendix 1 p 7). GPSC10, GPSC16, and GPSC206 are globally spreading lineages, and GPSC150 was only detected in west Africa, except for three isolates from Israel. Among these most common lineages, only GPSC10 was multidrug resistant, with most isolates exhibiting resistance to penicillin, co-trimoxazole, erythromycin, and tetracycline (appendix 1 p 8).

Phylogenetic analysis of the serotype 24F *cps* revealed a strong clonal but not geographical structure (appendix 1 p 9). The *cps* gene belonging to the same GPSC was clustered together regardless of the isolates' country of origin. This finding indicated the linkage between *cps* and its genetic background, and further confirmed that the emergence of serotype 24F in different geographical regions was due to clonal spread, rather than independent capsular switching. Only a few capsular switching events were observed based on the high similarity in *cps* between lineages. For example, three GPSC18 isolates from France share highly similar *cps* with GPSC10, suggesting a capsular switching between GPSC10 and GPSC18. GPSC18 expressing serotype 24F was not detected elsewhere but in France, according to the GPS project database and PubMLST database (last accessed on Aug 12, 2021). GPSC18-24F was first detected in 2011 in this collection of isolates, a year after the introduction of PCV13 in France, without statistically significant expansion (figure 1).

Of all serotype 24F lineages, GPSC10 was the only one detected with high invasive disease potential and multidrug resistance. GPSC10 was responsible for the increase of serotype 24F in France, and one of the major lineages mediating the global spread of serotype 24F. We further investigated this lineage using an international collection of 888 GPSC10 isolates. This lineage was detected in 33 countries across Africa, Asia, Europe, North America, South America, and Oceania (appendix 1 p 10). GPSC10 expressed 17 different serotypes, including 3, 6A, 6C, 7B, 10A, 11A, 13, 14, 15B, 15C, 17F, 19A, 19F, 23A, 23B, 23F, and 24F. Only six of these serotypes (serotypes 3, 6A, 14, 19A, 19F, and 23F) are covered by the current PCVs (PCV10 and PCV13) approved for use in children. An additional three serotypes (10A, 11A, and 15B) are covered by Pfizer's planned 20-valent vaccine, and four (10A, 11A, 15B, and 17F) by Merck's planned 24-valent vaccine. These two vaccines had not been approved for use in children at the time of writing. At present, no single vaccine is known to cover all 17 serotypes expressed by GPSC10. Concerningly, GPSC10 is among the top five lineages in India,^31^ Pakistan, and Nepal (appendix 1 p 12), where pneumococcal disease burden is highest.^1^ In these three countries, 15 serotype variants were detected in GPSC10 and only six were included in PCV13, underlining the potential of GPSC10 to cause serotype replacement in the future. Globally, GPSC10 was consistently found to express multiple serotypes and be multidrug resistant.

**Figure 3:**
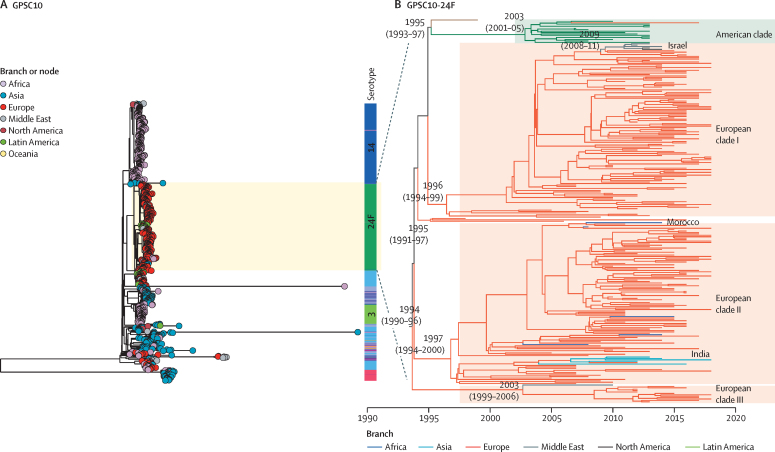
Global phylogeny of GPSC10 (A) and time-resolved phylogeny of a cluster of GPSC10-24F *Streptococcus pneumoniae* (B) All but one serotype 24F isolates were clustered together, regardless of their country of origin, indicating a clonal spread of GPSC10-24F across the world. The GPSC10 global phylogeny and time-resolved phylogeny can be interactively visualised in Microreact. GPSC=global pneumococcal sequence cluster.

The global GPSC10 phylogeny showed that all serotype 24F isolates except one were clustered together, regardless of their country of origin (figure 3A). This finding is consistent with the 24F *cps* phylogeny, which indicated that the global dissemination of GPSC10-24F was largely mediated by clonal spread. We analysed an additional collection of 91 GPSC10 isolates from Spain and detected a rapid switch in serotype composition from 19A to 24F, after the implementation of PCV13 in the target group of children (appendix 1 p 11). The 19A and 24F serotype variants from Spain were separated in long branches on the global phylogeny, further indicating that the capsular switch predates the vaccine introduction and coexistence of both GPSC10 serotype variants in Spain.

A time-resolved phylogeny was built on the cluster of 276 GPSC10-24F isolates and revealed four subclusters: EU-clade-I (dominated by ST4253), EU-clade-II (ST230 and ST4677), EU-clade-III (ST230), and American clade (ST230) (figure 3B). EU-clade-I and EU-clade-II were estimated to emerge around the 1990s and then clonally expand. EU-clade-III was relatively small and estimated to emerge around late 1990s to early 2000s, with majority of isolates from France, and one from Qatar. Throughout the study period, EU-clade-I accounted for most (112 [70%] of 160) of the French GPSC10-24F isolates and drove the 24F increase, and most of Spanish isolates (58 [88%] of 66) belonged to EU-clade II (appendix 1 p 12). In the EU-clade-I, long-range transmission was observed from Europe to Israel in the late 2000s. In the EU-clade-II, multiple transmissions from Europe to Morocco were detected, and a single transmission from Europe to India in the early 2000s was followed by a clonal expansion.

**Figure 4:**
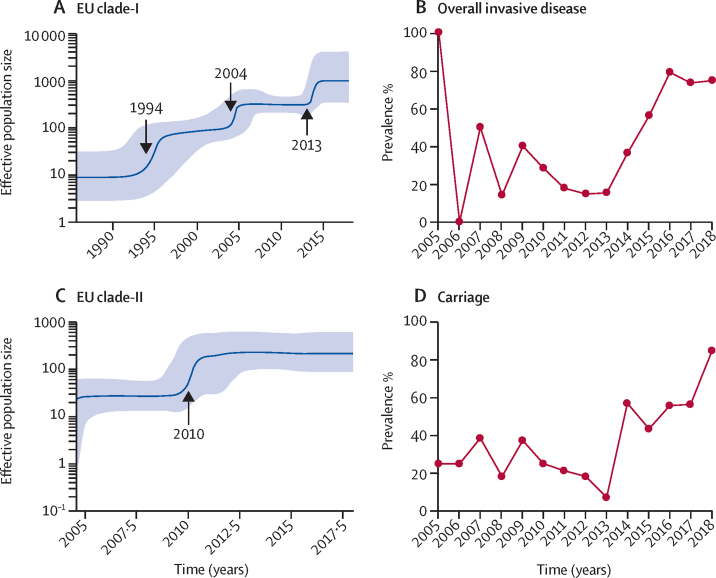
Bayesian skyline plots of estimated median effective population size of EU-clade-I and EU-clade II of GPSC10-24F *Streptococcus pneumoniae* over time (A, C) and observed prevalence of GPSC10-24F *S pneumoniae* from overall invasive disease and carriage in France over the collection year (B, D) The figure shows three exponential increases in effective population size in the EU-clade-I and one exponential increase in the EU-clade-II. The most recent increase in EU-clade-I coincided with the observed prevalence increase of GPSC10-24F in both overall disease and carriage isolates in France. GPSC=global pneumococcal sequence cluster.

We reconstructed the population dynamics over time on EU-clade-I and EU-clade-II using the Bayesian skyline model. The EU-clade-I was predicted to have three exponential increases in effective population size in around 1994, 2004, and 2013, and the EU-clade-II had one in around 2010 (figure 4A, C). The most recent increase in EU-clade-I coincided with the observed prevalence increase of GPSC10-24F in both overall disease and carriage isolates from France (figure 4A, B, D). Spatiotemporal analysis of the 174 GPSC10-24F isolates from France indicated that a pair of pneumococcal isolates had a higher risk ratio of being recovered within the same province if they were less than 5 years diverged from their most recent common ancestor (MRCA), compared with pairs that were 5 or more years diverged from their MRCA. Pairs which had diverged 5 or more years ago had risk ratio spanning 1, suggesting that the GPSC10-24F population was homogeneous across French provinces in 3–5 years (appendix 1 pp 13, 18).

## Discussion

Our results showed the emergence of the virulent and multidrug resistant pneumococcal lineage GPSC10 (CC230) that was responsible for the increase of invasive pneumococcal disease in France and the global spread of the invasive serotype 24F, which is not included in the current or upcoming PCVs. Due to its recombinogenic nature,^32^ this lineage is capable of simultaneously expressing a wide range of serotypes to facilitate its adaptation under the vaccine-selective pressure. Together with its transmissibility, GPSC10 should therefore be regarded as a high-risk lineage that could diminish the benefits of the vaccination programme worldwide over time.

Since the advent of the first PCV in 2000, GPSC10 has mediated serotype replacement in multiple countries. After the introduction of PCV7 and PCV10, 19A became the predominant serotype causing invasive disease in Europe; this increase was partially driven by GPSC10, together with GPSC1 (CC320).^33^, ^34^, ^35^, ^36^, ^37^, ^38^ In France and Spain, the major contributor of serotype 19A was GPSC10 in the post-PCV7 period.^39^ The use of PCV13 (which targets 19A) effectively reduced serotype 19A but with a concurrent increase in serotype 24F in Europe.^2^, ^10^, ^17^, ^19^, ^40^ Using the Spanish collection, we observed a rapid change in serotype composition within GPSC10 from 19A to 24F after PCV13. A similar serotype change within GPSC10 was also observed in Argentina^5^ and Israel^41^ after PCV13, demonstrating that the pre-existence of serotype variants enabled GPSC10 to survive and expand under the vaccine-selective pressure.

Serotype 24F pneumococci were infrequent causes of invasive disease in Europe in the 1980s,^42^ and the earliest detection of the GPSC10 lineage was an isolate from Denmark in 1996, which expressed serotype 14 (pubMLST, strain M132-14). GPSC10-24F was first reported from three adult patients in Naples, Italy between 1997 and 1998;^42^ these findings coincide with our model prediction of the emergence of this clone in Europe. Since the late 1990s, GPSC10-24F has become more frequently detected in children^2^, ^33^ and adults^31^, ^43^ from southern Europe. Despite the geographical proximity between France and Spain, the increase in serotype 24F was mainly driven by two different clones, GPSC10 EU-clade-I in France and EU-clade-II in Spain. This finding might suggest trans-border transmission is present, but not as frequently as transmission within a country, resulting in evolution of two clones in parallel.

Although the global spread of serotype 24F is largely due to the clonal spread of three pneumococcal lineages (GPSC10, GPSC16, and GPSC206), the lineage driving the increase in serotype 24F differs between countries. This variation could be partially explained by the differences in antibiotic-selective pressure. For example, the 24F driver lineage in Denmark was GPSC6.^7^ Unlike GPSC10, GPSC6 was susceptible to penicillin and erythromycin. This observation coincided with the lower consumption of penicillin and macrolides (a class of antibiotic that includes erythromycin) in Denmark, as compared with other countries such as France,^2^, ^3^ Lebanon,^12^ and Spain^14^, ^15^ where the increase in serotype 24F was mediated by the multidrug-resistant GPSC10 (appendix 1 p 23) lineage. According to ResistanceMap, France, Lebanon, and Spain consumed 1·8–2·2 times more penicillin and 1·2–2·0 times more macrolides than Denmark in 2015. GPSC10 also mediated the increase in serotype 24F in Argentina,^5^ where macrolide consumption was 1·2 times higher than in Denmark in 2015, even though penicillin consumption was similar. The high consumption of penicillin or erythromycin, or both, in Argentina, France, Lebanon, and Spain potentially selected for GPSC10, and serotype replacement in low antibiotic consumption settings was mainly observed to be mediated by susceptible lineages.^7^, ^44^ Among countries with an increase in serotype 24F, Japan has the highest consumption of erythromycin and the least consumption of penicillin, and the driver lineage in Japan, GPSC106 (CC2572), only exhibits erythromycin resistance.^11^ These observations suggest that vaccine-selective and antibiotic-selective pressure are shaping the post-vaccine population structure, and that the reduction in antibiotic resistance achieved by PCVs might gradually diminish as a result.^41^ However, resistance alone was not sufficient for clonal expansion, as GPSC10-24F was detected in all six countries but only expanded in settings with high antibiotic use. This finding echoed similar observations in other bacterial species such as *Escherichia coli*.^45^ Because acquired antibiotic-resistant genes are part of the accessory genome (genes not present in all isolates of a species), their frequencies are under negative-frequency dependent selection,^46^ which might explain the coexistence of susceptible and resistant strains in the pneumococcal population.

The limitations of this study were that the sampling strategy and collection timeframe were not consistent between countries, and the relatively small sample size after stratifications (eg, carriage and IPD). However, the GPS project, which was formulated to create a pre-PCV and post-PCV dataset in each participating country to evaluate the effect of PCVs on the pneumococcal population, not only provided a large global collection of pneumococcal genomes, but also a comprehensive set of epidemiological metadata. This unprecedented database allowed us to complement knowledge we gained from a collection of country-specific analyses to this overarching study, providing an international perspective on GPSC10 and highlighting this lineage as carrying a high future risk for pneumococcal disease requiring targeted prevention. This study also underlines the need for policy makers to evaluate the overall effect of PCVs including changes in IPD incidence and detection of emerging non-vaccine serotypes. Focusing only on vaccine serotypes in estimating effect could be misleading as it would identify a rise in serotype 19A, observed in some settings with PCV10 use, but would not detect the increase in serotype 24F, associated with PCV13 use. Knowledge gained through implementation of an effective and sustainable surveillance system for *S pneumoniae* could guide timely policy making including choice of PCV to stabilise the incidence of IPD cases at a low level or even further reduce incidence.

Our work further illustrates the usefulness of bacterial genome sequencing to better understand the pneumococcal lineages behind serotype changes and reveals that GPSC10 alone is a challenge for a serotype-based vaccine strategy. More systematic investigation to identify lineages like GPSC10 will better inform and improve next-generation preventive strategies against pneumococcal disease.

## Data sharing

All sequencing reads were deposited in the European Nucleotide Archive (https://www.ebi.ac.uk/ena/browser/home) and the accession numbers are with the metadata and in silico output in appendix 2.

## Declaration of interests

AvG reports grants from the US Centers for Disease Control and Prevention and Sanofi; travel fees from Pfizer, Sanofi, and Merck; and is the chairperson of South Africa National Advisory Group on Immunisation, outside of the submitted work. AJP reports grants from Gavi, WHO, and AstraZeneca, and is a member of the Joint Committee on Vaccination and Immunisation and WHO Strategic Advisory Group of Experts on Immunization, outside of the submitted work. IN reports grants from BMGF and travel fees from the International Symposium on Pneumococci and Pneumococcal Diseases, outside of the submitted work. JM is an employee of Pharma Vaccines, outside of the submitted work. RD reports grants from Pfizer, MSD, and MedImmune–AstraZeneca, consulting and speaker fees from Pfizer and MSD, outside of the submitted work. SDB reports personal fees from Pfizer and Merck, outside the submitted work. CMA reports grants from Pfizer, outside of the submitted work. EV report grants from Santé Publique France, Pfizer, and MSD, outside of the submitted work. All other authors declare no competing interests.
