# Supplementary material for: Emergence of a multidrug-resistant and virulent Streptococcus pneumoniae lineage mediates serotype replacement after PCV13: an international whole-genome sequencing study
Source: Lancet Microbe. 2022 Oct;3(10):e735–43. doi: 10.1016/S2666-5247(22)00158-6 (PMC9519462; doi:10.1016/S2666-5247(22)00158-6)
Supplement: Supplementary appendix 1 [file mmc1.pdf]

THE LANCET
Microbe

## Supplementary appendix 1

This appendix formed part of the original submission and has been peer reviewed. We post it as supplied by the authors.

Supplement to: Lo S W, Mellor K, Cohen R, et al. Emergence of a multidrug-resistant and virulent Streptococcus pneumoniae lineage mediates serotype replacement after PCV13: an international whole-genome sequencing study. *Lancet Microbe* 2022; published online August 16. https://doi.org/10.1016/S2666-5247(22)00158-6.

**Supplementary appendix**

This appendix formed part of the original submission and has been peer reviewed.
We post it as supplied by the authors.

Supplement to: Lo S, Mellor K, Cohen R et al. Emergence of a multidrug-resistant and virulent Streptococcus pneumoniae lineage mediates serotype replacement after PCV13: an international whole-genome sequencing study. *Lancet Microbe*

**APPENDIX**

**Supplementary Methods** ……………………………………………………………………………………………4-5

**Supplementary Figures**

Figure S1. A collection of serotype 24F *Streptococcus pneumoniae* from France 2003-2018 by clinical sample source, age in years and year of collection. ………………………..6

Figure S2. The geographical distribution of serotype 24F *Streptococcus pneumoniae* (n=642) in the Global Pneumococcal Sequencing (GPS) project database, including 419 isolates from France. ……………………………………………………………………………………..7

Figure S3. Phylogeny of 642 serotype 24F Streptococcus pneumoniae from 29 countries across six continents overlaid with antibiotic resistance profiles. …………….8

Figure S4. A phylogeny built upon the genetic variants identified from the capsular encoding region (cps) in a collection of 642 serotype 24F Streptococcus pneumoniae and overlaid with Global Pneumococcal Sequence Clusters (GPSCs). ………………………9

Figure S5. Geographical distribution of Global Pneumococcal Sequence Cluster (GPSC)10 (n=888) from 33 countries. ………………………………………………………………….10

Figure S6. Rapid changes in serotype composition within Global Pneumococcal Sequence Cluster (GPSC)10 during PCV introductions among 91 isolates from Spain. ………………………………………………………………………………………………………….11

Figure S7. Figure S7. The proportion of Global Pneumococcal Sequencing Cluster (GPSC)10 clades and other GPSCs in serotype 24F Streptococcus pneumoniae isolates causing invasive pneumococcal disease from France and Spain. ……………………………12

Figure S8. Spatiotemporal analysis of GPSC10-24F sub-lineage from France. …………13

**Supplementary Tables**

Table S1. Prevalence of GPSCs in serotype 24F Streptococcus pneumoniae causing invasive disease (n=190) and asymptomatic colonisation (n=229) in France over vaccine periods. …………………………………………………………………………………………………..14

Table S2. Difference in Global Pneumococcal Sequence Cluster (GPSC) between children aged <5 and individuals aged 5-17years old in France. …………………………….15

Table S3. Table S3. Difference in sequence type (ST) between children aged <5 and individuals aged 5-17years old in France. ………………………………………………………….16

Table S4. Difference in antimicrobial resistance between children aged <5 and individuals aged 5-17years old in France. ………………………………………………………….17

Table S5 Odds ratio for invasiveness and propensity to cause meningitis of six pneumococcal lineages expressing serotype 24F from France. ……………………………..18
…
Table S6 A pairwise risk ratio for two samples being diverged from time-to-most-recent-common-ancestor (tMRCA) and recovered from the same French province. 19

Table S7 The prevalence, serotypes and resistance profile of GPSC10 by country in the Global Pneumococcal Sequencing (GPS) database. ……………………………………………20-22

Table S8. The relationship between pneumococcal 24F driver lineages and antibiotic consumption. ……………………………………………………………………………………..…………………….23

Supplementary Methods

The -80°C stock of each *S. pneumoniae* isolate was plated on an agar plate with 5% sheep blood and incubated overnight at 37°C in 5% CO2. A single colony from the overnight culture was inoculated in 5ml Todd Hewitt broth at 37°C in 5% $CO_2$ overnight. The bacterial pellet from the overnight broth culture was then subject to DNA extraction. Pneumococcal DNA was extracted using a modified protocol of QIAamp1DNAMini Kit (QIAGEN, IncValencia, CA) protocol as previously described [1]. The DNA quantity was evaluated by Qubit and then subject to sequencing on an Illumina HiSeq platform at Wellcome Sanger Institute, generating ≥100bp paired-end reads. The reads were assembled and annotated as previously described.[2] Quality control of the genome sequences was as follow: 1) overall sequencing depth >20X, 2) >60% reads mapping to Streptococcus pneumoniae using Kraken, 3) >60% mapping coverage of reference genome (PMEN global clone Spain23F-1, accession number FM211187) 4) percent of heterozygous sites over total number of single nucleotide polymorphisms (SNPs) ≤ 15%, 5) total number of contigs <500 and 6) total length of the assembled genome size between 1.9-2.3 Mb. Serotypes were predicted from the sequence reads using SeroBA.[3] At the time of writing, SeroBA could not differentiate serotypes within serogroup 24. Therefore, serogroup 24 isolates (n=674) in the Global Pneumococcal Sequencing (GPS) project, including those from France, identified by SeroBA were subject to phylogenetic analysis. Reads of serogroup 24 genomes were mapped to the reference sequence of 24F capsular encoding region *cps* (CR931688) using Burrows Wheeler Aligner (BWA) version 0.7.17-r1188.[4] The alignment was then further aligned with reference sequences of 24A (CR931686) and 24B (CR931687), followed by extracting SNPs using snp-sites.[5] A maximum likelihood tree using FastTree version 2.1.10[6] with GTR substitution model was constructed and overlaid with phenotypic serotyping results if available. We identified 25 isolates clustered with 24A reference, 94 with serotype 24F reference, 549 in a group that 250 isolates were confirmed as 24F by the Quellung reaction in eight different laboratories, and two divergent isolates. No isolate's *cps* was clustered with serotype 24B reference. The 642 isolates predicted to be serotype 24F were included for further analysis.

References

1 Hawkins PA, Akpaka PE, Nurse-Lucas M, *et al.* Antimicrobial resistance determinants and susceptibility profiles of pneumococcal isolates recovered in Trinidad and Tobago. *Journal of Global Antimicrobial Resistance* 2017; published online Aug 14. DOI:10.1016/j.jgar.2017.08.004.

2 Page AJ, De Silva N, Hunt M, *et al.* Robust high-throughput prokaryote de novo assembly and improvement pipeline for Illumina data. *Microb Genom* 2016; **2**: e000083.

3 Epping L, van Tonder AJ, Gladstone RA, *et al.* SeroBA: rapid high-throughput serotyping of Streptococcus pneumoniae from whole genome sequence data. *Microb Genom* 2018; **4**. DOI:10.1099/mgen.0.000186.

4 Li H, Durbin R. Fast and accurate short read alignment with Burrows-Wheeler transform. *Bioinformatics* 2009; **25**: 1754–60.

5 Page AJ, Taylor B, Delaney AJ, *et al.* SNP-sites: rapid efficient extraction of SNPs from multi-FASTA alignments. *Microb Genom* 2016; **2**: e000056.

6 Price MN, Dehal PS, Arkin AP. FastTree 2--approximately maximum-likelihood trees for large alignments. *PLoS One* 2010; **5**: e9490.

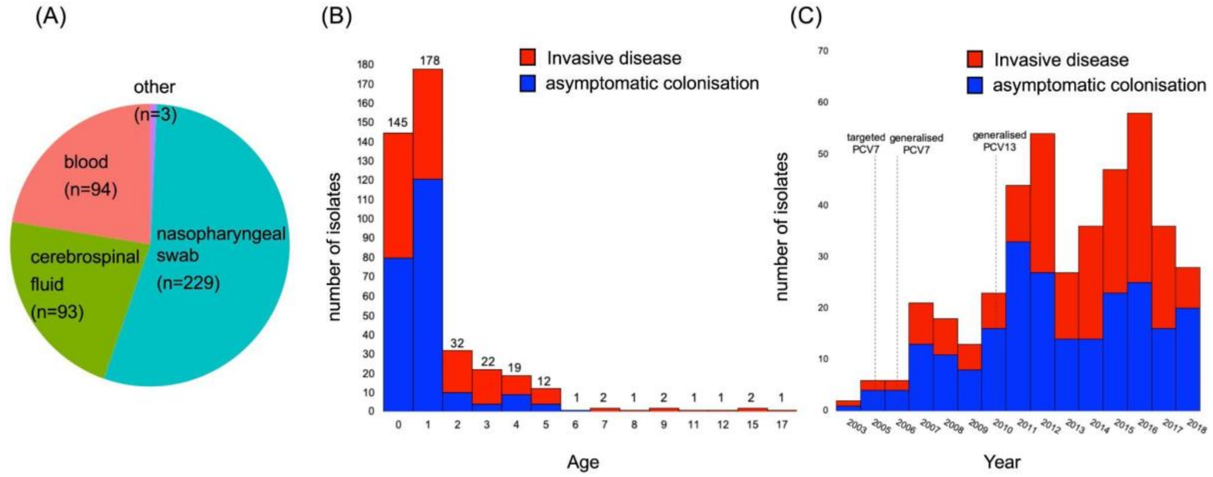


Figure S1 A collection of serotype 24F *Streptococcus pneumoniae* from France 2003-2018 by (A) clinical sample source, (B) age in years and (C) year of collection. The collection indicates an almost 1:1 ratio of samples from invasive disease (cerebrospinal fluid, blood, and others) and asymptomatic colonisation (nasopharyngeal swab) overall and over the year. Majority of samples are from children aged 2 years or less.

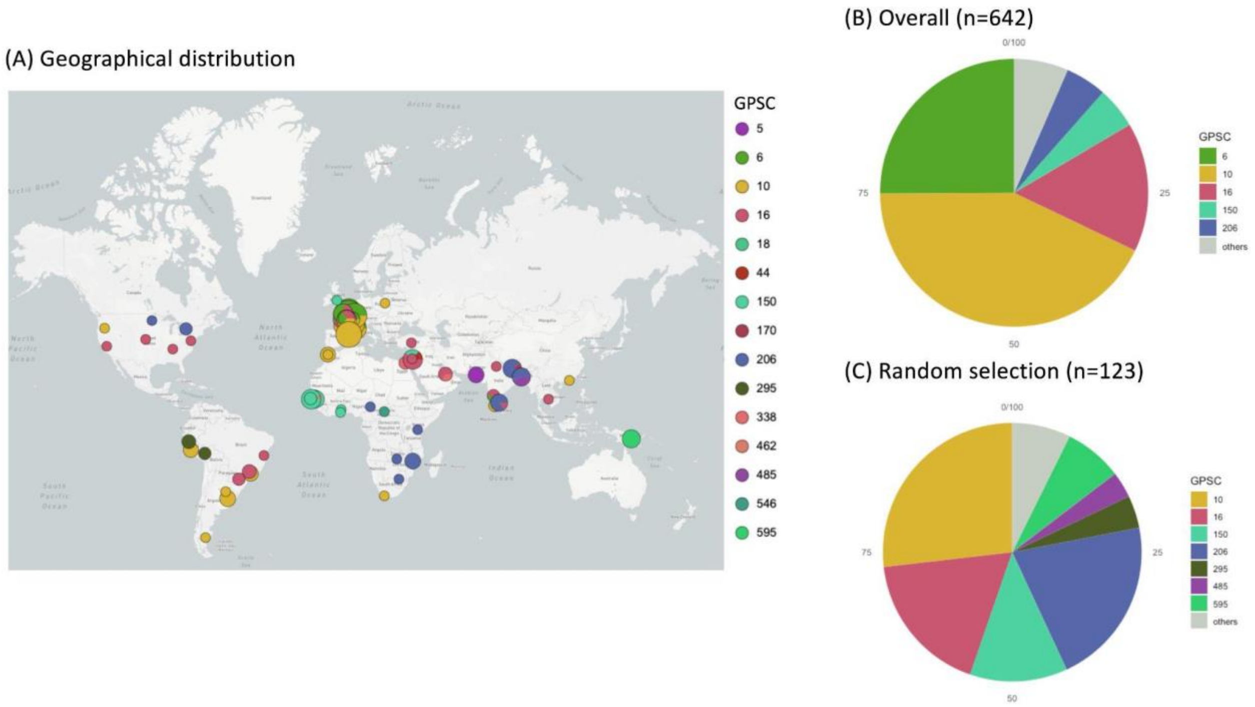


Figure S2 (A)The geographical distribution of serotype 24F *Streptococcus pneumoniae* (n=642) in the Global Pneumococcal Sequencing (GPS) project database, including 419 isolates from France. (B) Proportion of pneumococcal lineages or Global Pneumococcal Sequence Clusters (GPSCs) in an overall collection of serotype 24F pneumococci (n=642) and a sub-collection (n=123) includes isolates randomly selected from disease surveillance systems and carriage surveys. Pneumococcal lineages less than 3% in prevalence are grouped as others in the pie charts. The geographical distribution can be interactively visualised at https://microreact.org/project/global_24F/7d36573f .

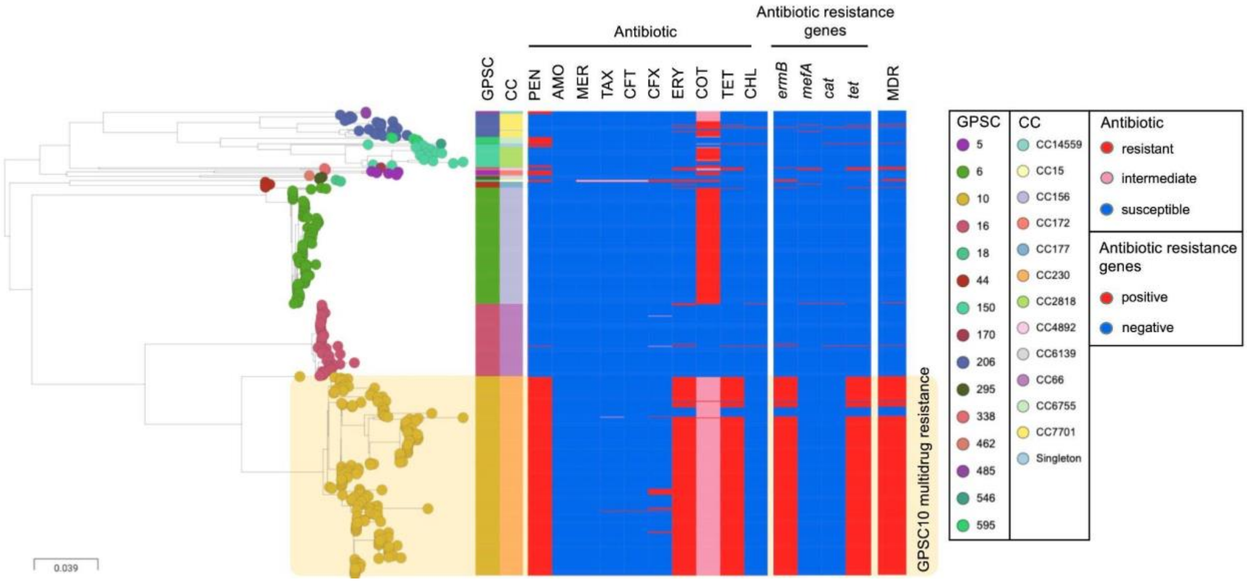


Figure S3 Phylogeny of 642 serotype 24F *Streptococcus pneumoniae* from 29 countries across six continents overlaid with antibiotic resistance profiles.
GPSC, global pneumococcal sequence cluster; CC, clonal complex; PEN, penicillin; AMO, amoxicillin; MER, meropenem; TAX, cefotaxime; CFT, ceftriaxone; CFX, cefuroxime; ERY, erythromycin; COT, cotrimoxazole; TET, tetracycline; CHL, chloramphenicol; MDR, multidrug resistance. This figure can be interactively visualised at https://microreact.org/project/global_24F/e1acf229 .

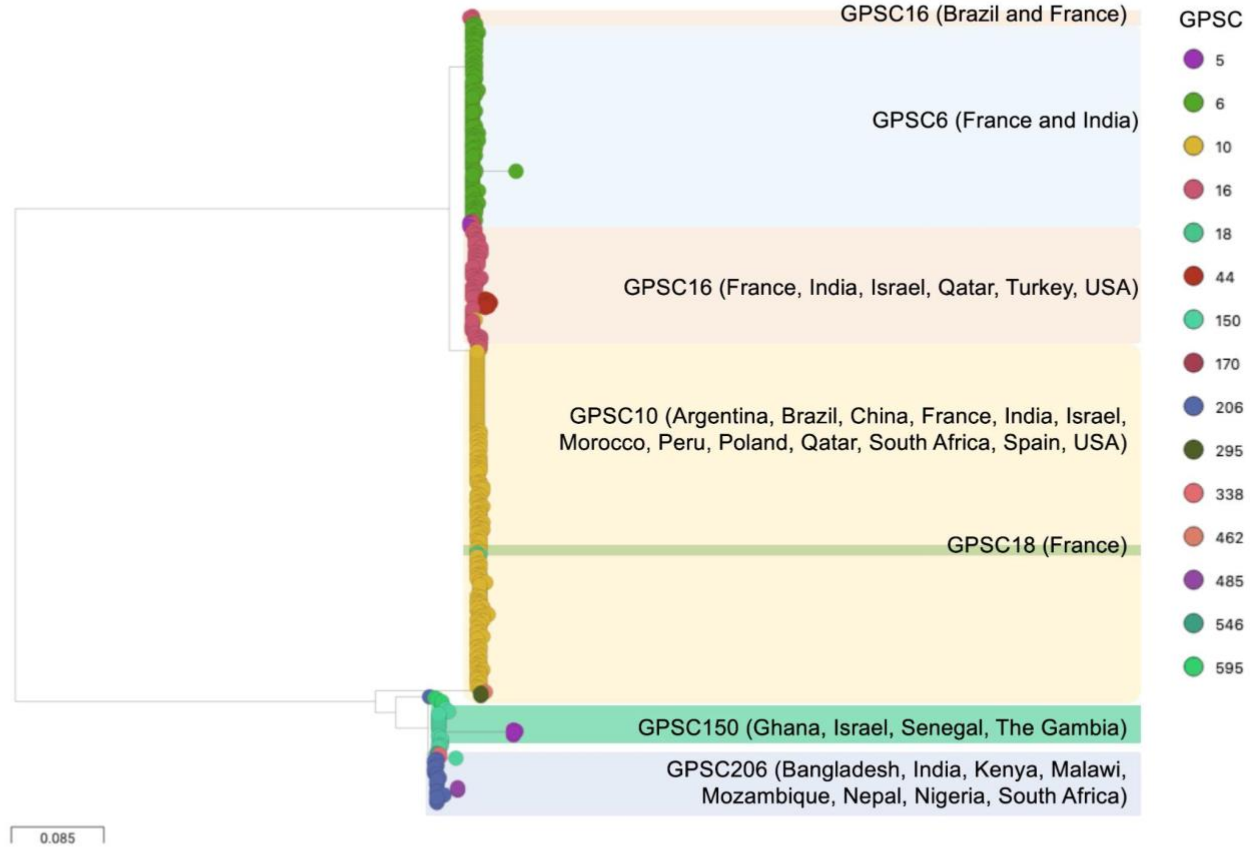


Figure S4 A phylogeny built upon the genetic variants identified from the capsular encoding region (*cps*) in a collection of 642 serotype 24F *Streptococcus pneumoniae* and overlaid with Global Pneumococcal Sequence Clusters (GPSCs)

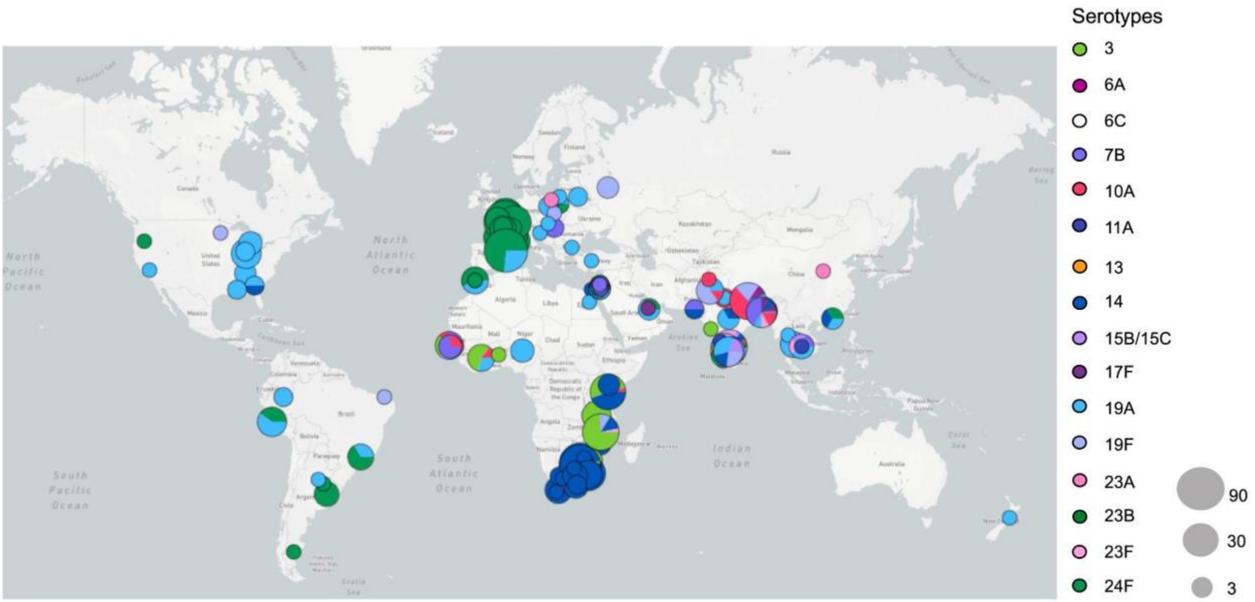


Figure S5 Geographical distribution of Global Pneumococcal Sequence Cluster (GPSC)10 (n=888) from 33 countries. This figure can be interactively viewed at https://microreact.org/project/global_GPSC10/21948517

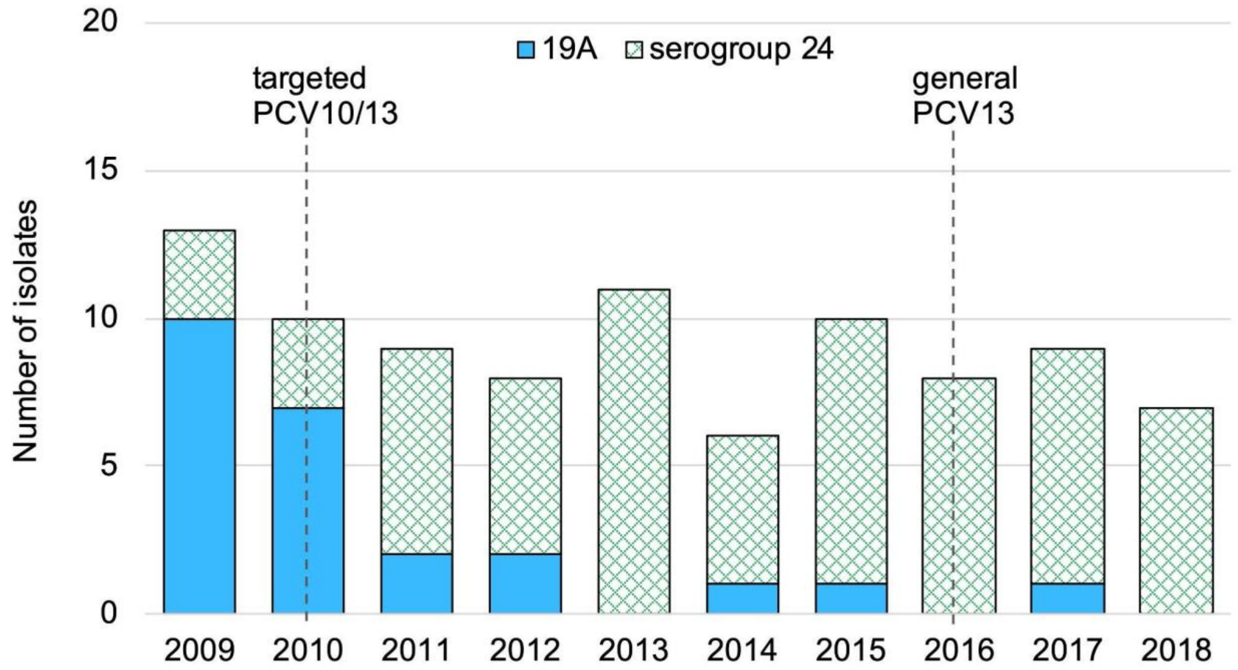

Figure S6. Rapid changes in serotype composition within Global Pneumococcal Sequence Cluster (GPSC)10 during PCV introductions among 91 isolates from Spain. Serotype 19A is targeted by PCV13 but not PCV10. Serotype 24F is not targeted by either PCV.

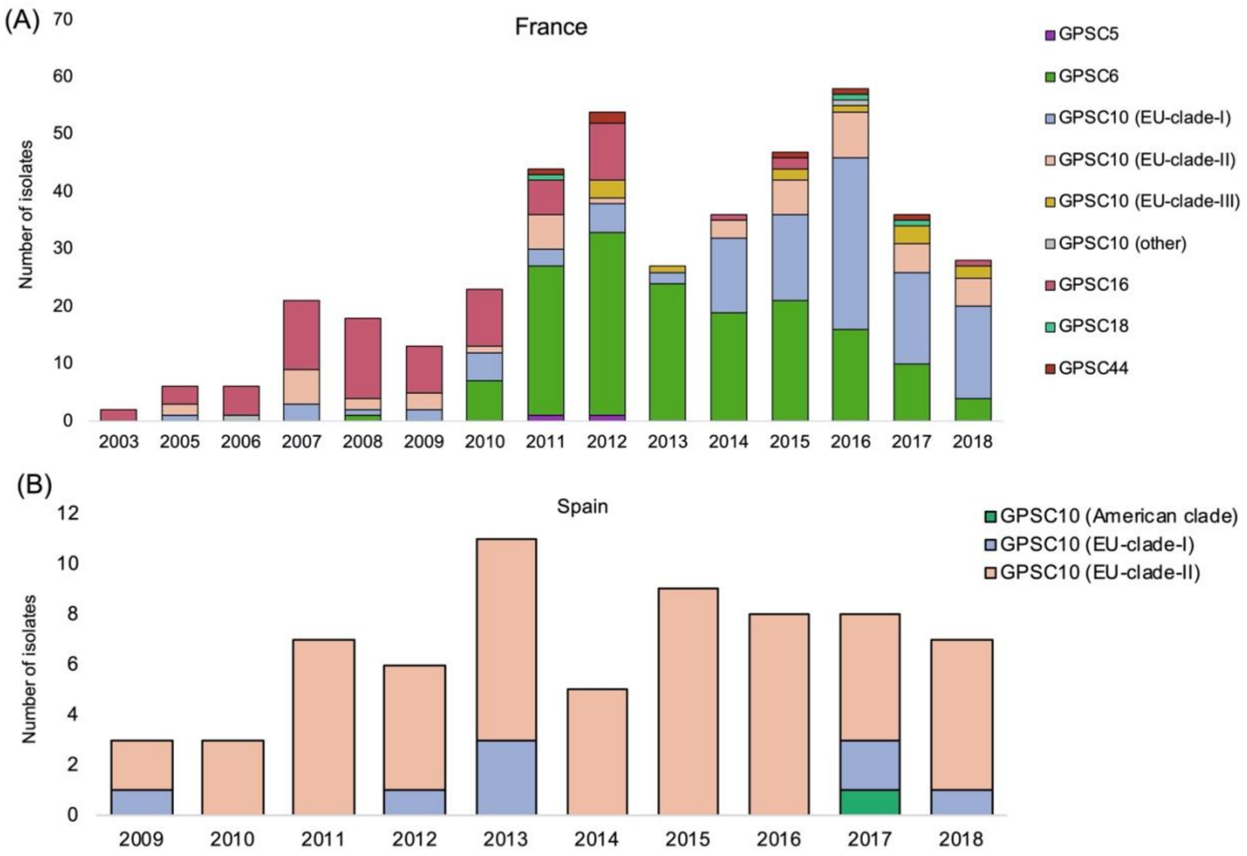


Figure S7. The proportion of Global Pneumococcal Sequencing Cluster (GPSC)10 clades and other GPSCs in serotype 24F *Streptococcus pneumoniae* isolates causing invasive pneumococcal disease from France (A) and Spain (B).

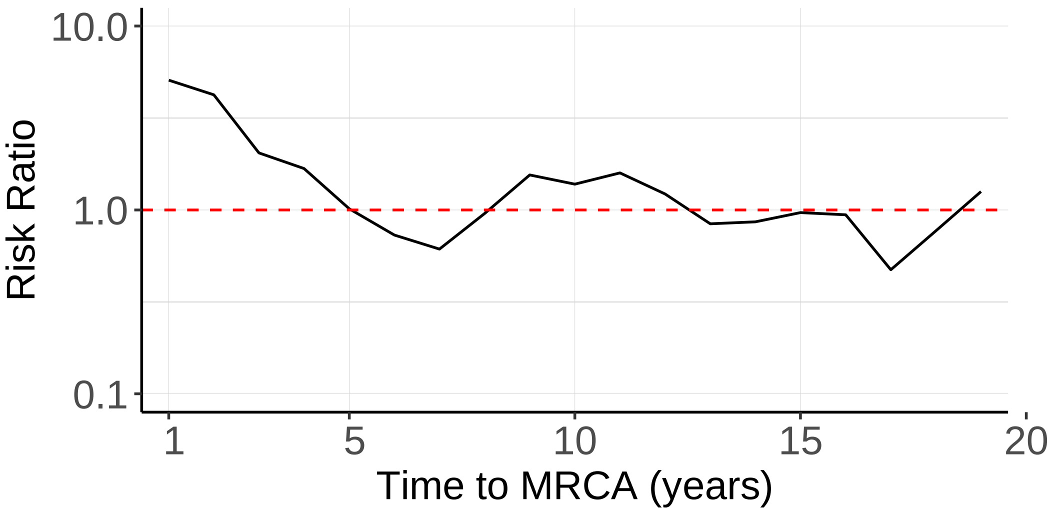

Figure S8 Spatiotemporal analysis of GPSC10-24F sub-lineage from France. A pairwise odds ratio was calculated for two samples being diverged from time-to-most-recent-common-ancestor (tMRCA) and recovered from the same French province. Risk ratio higher than one indicates that a pair of isolates is more likely to be recovered within the same French province. Pairs diverged 5 or more years ago had a risk ratio ~ 1 without up- or downward trend, indicating an equal chance to recover the sample pair within and between French provinces.

Table S1 Prevalence of GPSCs in serotype 24F *Streptococcus pneumoniae* causing invasive disease (n=190) and asymptomatic colonisation (n=229) in France over vaccine periods.

| | n(%) | | | | P value | | |
|---|---|---|---|---|---|---|---|
| Lineages | Targeted PCV7 (2003-2005) | Generalised PCV7 (2006-2010) | Early PCV13 (2011-2014) | Late PCV13 (2015-2018) | Targeted PCV7 vs generalised | Generalised PCV7 vs Early PCV13 | Early PCV13 vs Late PCV13 |
| GPSC5 | - | - | 2 (1.2) | - | - | - | - |
| GPSC6 | - | 8 (9.9) | 101 (62.7) | 51 (30.2) | - | <0.05* | <0.05* |
| GPSC10 | 3 (37.5) | 24 (29.6) | 37 (23.0) | 110 (65.1) | 1.00 | 0.63 | <0.05* |
| GPSC16 | 5 (62.5) | 49 (60.5) | 17 (10.6) | 3 (1.8) | 1.00 | <0.05* | <0.05* |
| GPSC18 | - | - | 1 (0.6) | 2 (1.2) | - | - | 1.00 |
| GPSC44 | - | - | 3 (1.9) | 3 (1.8) | - | - | 1.00 |

*Asterisk next to the p value indicates statistical significance.

Table S2. Difference in Global Pneumococcal Sequence Cluster (GPSC) between children aged <5 and individuals aged 5-17years old in France

| | Number of isolates (%) | | | |
|---|---|---|---|---|
| IPD | Age | | | |
| GPSC | <5 yr | ≥5 yr | P value | Adjusted p value[a] |
| 6 | 60 (35) | 10 (56) | 0.12 | 0.34 |
| 10 | 82 (48) | 5 (28) | 0.14 | 0.34 |
| 16 | 28 (16) | 3 (16) | 1.00 | 1.00 |
| 18 | 1 (0.5) | - | 1.00 | 1.00 |
| 44 | 1 (0.5) | - | 1.00 | 1.00 |
| | | | | |
| Carriage | | | | |
| 5 | 2 (0.9) | 0 | 1.00 | 1.00 |
| 6 | 89 (40) | 1 (20) | 0.65 | 1.00 |
| 10 | 83 (37) | 4 (80) | 0.07 | 0.42 |
| 16 | 43 (19) | 0 | 0.59 | 1.00 |
| 18 | 2 (0.9) | 0 | 1.00 | 1.00 |
| 44 | 5 (2) | 0 | 1.00 | 1.00 |

[a]P value was adjusted for multiple testing correction using the Benjamini-Hochberg false discovery rate of 5%.

Table S3. Difference in sequence type (ST) between children aged <5 and individuals aged 5-17years old in France

| | IPD no. of isolates (%) | | | | | Carriage no. of isolates (%) | | | |
|---|---|---|---|---|---|---|---|---|---|
| | Age | | | | | Age | | | |
| GPSC/ST | <5 yr (n=172) | ≥5 yr (n=18) | P value | Adjusted p value [a] | | <5 yr (n=224) | ≥5 yr (n=5) | P value | Adjusted p value[a] |
| **GPSC5** | | | | | | | | | |
| ST338 | - | - | - | - | | 2 (0.9) | - | 1.00 | 1.00 |
| | | | | | | | | | |
| **GPSC6** | | | | | | | | | |
| ST162 | 60 (35) | 10 (56) | 0.12 | 1.00 | | 88 (39) | 1 (20) | 0.65 | 1.00 |
| ST15752 | - | - | - | - | | 1 (0.4) | - | 1.00 | 1.00 |
| | | | | | | | | | |
| **GPSC10** | | | | | | | | | |
| ST230 | 22 (13) | 2 (10) | 1.00 | 1.00 | | 26 (12) | - | 1.00 | 1.00 |
| ST4253 | 44 (26) | 3 (17) | 0.57 | 1.00 | | 47 (21) | 4 (80) | <0.05* | 0.16 |
| ST4266 | 1 (0.5) | - | 1.00 | 1.00 | | | | | |
| ST4677 | 7 (4) | - | 1.00 | 1.00 | | 4 (2) | - | 1.00 | 1.00 |
| ST6227 | 1 (0.5) | - | 1.00 | 1.00 | | 2 (0.9) | - | 1.00 | 1.00 |
| ST8857 | 1 (0.5) | - | 1.00 | 1.00 | | | | | |
| ST10921 | - | - | - | - | | 1 (0.4) | - | 1.00 | 1.00 |
| ST15584 | 1 (0.5) | - | 1.00 | 1.00 | | - | - | - | - |
| ST15585 | 1 (0.5) | - | 1.00 | 1.00 | | - | - | - | - |
| ST15586 | 1 (0.5) | - | 1.00 | 1.00 | | - | - | - | - |
| ST15587 | 1 (0.5) | - | 1.00 | 1.00 | | 1 (0.4) | - | 1.00 | 1.00 |
| ST15755 | - | - | - | - | | 1 (0.4) | - | 1.00 | 1.00 |
| ST15759 | 1 (0.5) | - | 1.00 | 1.00 | | - | - | - | - |
| ST15767 | 1 (0.5) | - | 1.00 | 1.00 | | 1 (0.4) | - | 1.00 | 1.00 |
| | | | | | | | | | |
| **GPSC16** | | | | | | | | | |
| ST72 | 27 (16) | 3 (17) | 1.00 | 1.00 | | 41 (18) | - | 0.59 | 1.00 |
| ST9770 | 1 (0.5) | - | 1.00 | 1.00 | | | | | |
| ST13939 | - | - | - | - | | 1 (0.4) | - | 1.00 | 1.00 |
| ST15742 | - | - | - | - | | 1 (0.4) | - | 1.00 | 1.00 |
| | | | | | | | | | |
| **GPSC18** | | | | | | | | | |
| ST15 | 1 (0.5) | - | 1.00 | 1.00 | | 1 (0.4) | - | 1.00 | 1.00 |
| ST15751 | - | - | - | - | | 1 (0.4) | - | 1.00 | 1.00 |
| | | | | | | | | | |
| **GPSC44** | | | | | | | | | |
| ST177 | 1 (0.5) | - | 1.00 | 1.00 | | 5 (2) | - | 1.00 | 1.00 |

[a]p value was adjusted for multiple testing correction using the Benjamini-Hochberg false discovery rate of 5%.

*Asterisk next to the p value indicates statistical significance.

Table S4. Difference in antimicrobial resistance between children aged <5 and individuals aged 5-17years old in France

| | IPD no. of isolates (%) | | | | | Carriage no. of isolates (%) | | | |
|---|---|---|---|---|---|---|---|---|---|
| | Age | | | | | Age | | | |
| Penicillin | <5 yr | ≥5 yr | p value | Adjusted p value[b] | | <5 yr | ≥5 yr | p value | Adjusted p value[b] |
| Resistance | 83 (48) | 5 (28) | 0.14 | 0.47 | | 87 (39) | 4 (80) | 0.08 | 0.12 |
| Susceptible | 89 (52) | 13 (72) | | | | 137 (61) | 1 (20) | | |
| | | | | | | | | | |
| Chloramphenicol | | | | | | | | | |
| Resistance | 0 | 0 | 1.00 | 1.00 | | 1 (0.4) | 0 | 1.00 | 1.00 |
| Susceptible | 172 (100) | 18 (100) | | | | 223 (99.6) | 5 (100) | | |
| | | | | | | | | | |
| Erythromycin | | | | | | | | | |
| Resistance | 74 (43) | 5 (28) | 0.31 | 0.47 | | 87 (39) | 4 (80) | 0.08 | 0.12 |
| Susceptible | 98 (57) | 13 (72) | | | | 137 (61) | 1 (20) | | |
| | | | | | | | | | |
| Cotrimoxazole | | | | | | | | | |
| Non-susceptible[a] | 143 (83) | 15 (83) | 1.00 | 1.00 | | 174 (78) | 5 (100) | 0.59 | 0.71 |
| Susceptible | 29 (17) | 3 (17) | | | | 50 (22) | 0 | | |
| | | | | | | | | | |
| Tetracycline | | | | | | | | | |
| Resistance | 73 (42) | 5 (28) | 0.32 | 0.47 | | 82 (37) | 4 (80) | 0.07 | 0.12 |
| Susceptible | 99 (58) | 13 (72) | | | | 142 (63) | 1 (20) | | |
| | | | | | | | | | |
| Multidrug resistance | | | | | | | | | |
| Yes | 74 (43) | 5 (28) | 0.31 | 0.47 | | 85 (38) | 4 (80) | 0.08 | 0.12 |
| No | 98 (57) | 13 (72) | | | | 139 (62) | 1 (20) | | |

[a]Pneumococcal isolates predicted to be intermediate or fully resistant to cotrimoxazole are categorised as non-susceptible.
[b] P value was adjusted for multiple testing correction using the Benjamini-Hochberg false discovery rate of 5

Table S5 Odds ratio for invasiveness and propensity to cause meningitis of six pneumococcal lineages expressing serotype 24F from France.

| | % (n) | | | Overall disease vs carriage | | Meningitis vs Carriage | |
|---|---|---|---|---|---|---|---|
| GPSC | Overall disease (n=190) | Meningitis (n=102) | Carriage (n=229) | Odds ratio | p value | Odds ratio | p value |
| GPSC5 | 0 | 0 | 2 | 0 (0-Inf) | 0.20 | 0 (0-Inf) | 0.34 |
| GPSC6 | 37% (70) | 27% (28) | 39% (90) | 0.90 (0.61-1.34) | 0.61 | 0.58 (0.35-0.97) | 0.04 |
| GPSC10 | 46% (87) | 49% (50) | 38% (87) | 1.38 (0.93-2.04) | 0.11 | 1.57 (0.98-2.51) | 0.06 |
| GPSC16 | 16% (31) | 22% (22) | 19% (43) | 0.84 (0.51-1.40) | 0.51 | 1.19 (0.67-2.12) | 0.56 |
| GPSC18 | 0.5% (1) | 1% (1) | 0.9% (2) | 0.60 (0.05, 6.67) | 0.68 | 1.12 (0.10-12.54) | 0.92 |
| GPSC44 | 0.5% (1) | 1% (1) | 2% (5) | 0.24 (0.03, 2.05) | 0.16 | 0.44 (0.05-3.85) | 0.45 |

Table S6 A pairwise risk ratio for two samples being diverged from time-to-most-recent-common-ancestor (tMRCA) and recovered from the same French province.

| Divergence time window (tMRCA in years)[a] | Median divergence time window (tMRCA in years) | Risk ratio | lower 95% confidence interval | upper 95% confidence interval |
|---|---|---|---|---|
| 0 - 2 | 1 | 5.08 | 2.63 | 12.37 |
| 1 - 3 | 2 | 4.23 | 2.31 | 9.67 |
| 2 - 4 | 3 | 2.04 | 1.09 | 4.05 |
| 3 - 5 | 4 | 1.68 | 0.83 | 3.32 |
| 4 - 6 | 5 | 1.01 | 0.66 | 1.62 |
| 5 - 7 | 6 | 0.73 | 0.52 | 1.03 |
| 6 - 8 | 7 | 0.61 | 0.42 | 0.84 |
| 7 - 9 | 8 | 0.96 | 0.66 | 1.39 |
| 8 - 10 | 9 | 1.55 | 1.11 | 2.26 |
| 9 - 11 | 10 | 1.38 | 0.95 | 1.97 |
| 10 - 12 | 11 | 1.59 | 1.19 | 2.19 |
| 11 - 13 | 12 | 1.22 | 0.92 | 1.56 |
| 12 - 14 | 13 | 0.84 | 0.64 | 1.11 |
| 13 - 15 | 14 | 0.86 | 0.64 | 1.18 |
| 14 - 16 | 15 | 0.97 | 0.68 | 1.36 |
| 15 - 17 | 16 | 0.94 | 0.60 | 1.49 |
| 16 - 18 | 17 | 0.47 | 0.27 | 0.83 |
| 17 - 19 | 18 | 0.77 | 0.48 | 1.36 |
| 18 - 20 | 19 | 1.26 | 0.83 | 1.96 |

[a]The calculation of divergence time is based on a rolling window of two years.

Table S7 The prevalence, serotypes and resistance profile of GPSC10 by country in the Global Pneumococcal Sequencing (GPS) database

| | | | | GPSC10 | | | |
|---|---|---|---|---|---|---|---|
| Country/ Region | Sample type | n | Study period | rank | n | Serotypes (n) | Most common resistance profile |
| Argentina | Invasive disease | 413 | 1998-1999, 2010-2013 | 15th | 8 | 19A (1), 24F | PEN-COT-ERY-TET |
| Bangladesh | Invasive disease | 525 | 2002-2015 | 12th | 11 | 7B (4), 17F (1), 19A (2), 19F (3), 23F (1) | PEN-COT-ERY-TET |
| Brazil | Invasive disease | 466 | 2008-2009, 2012-2013 | 25th | 5 | 19A (3), 19F (1), 24F (1) | PEN-ERY-TET |
| Cambodia | Carriage | 686 | 2014-2017 | 22th | 5 | 7B(1), 11A (1), 15B/C (1), 19A (1), 23F (1) | PEN-COT-TET |
| Hong Kong (China) | Invasive disease (213) and Carriage (291) | 504 | 1995-2017 | 29th | 3 | 14(1), 19A(1), 24F(1) | PEN-COT-ERY-TET |
| India | Invasive disease (294) and Carriage (186) | 480 | 2009-2018 | 2nd | 47 | 3(1), 6A(3), 7B(2), 10A(5), 11A(1), 13(1), 14(1), 15/C(8), 19A(9), 19F(10), 22F(2), 24F(4) | PEN-COT-ERY-TET |
| Israel | Invasive disease | 1143 | 2005-2014 | 11th | 22 | 14(8), 17F(2), 19A(6), 19F(1), 23F(1), 24F(4) | PEN-COT-ERY-TET |
| Malawi | Invasive disease (555) and Carriage (749) | 1304 | 1997-2015 | 13th | 33 | 3(25), 14(4), 19F(3), 23F(1) | PEN-COT-TET |
| Mozambi | Invasive | 167 | 1996, | Rare | 1 | 14(1) | PEN-COT- |

| | | | | | | | |
|---|---|---|---|---|---|---|---|
| que | disease (167) | | 2008-2010 | | | | ERY-TET |
| Nepal | Invasive disease (93), Pneumonia* (245), Carriage (597) | 935 | 2005-2018 | 4th | 43 | 6A(1), 7B(11), 10A(17), 17F(2), 19A(3), 19F(9) | PEN-COT-ERY-TET |
| Nigeria | Invasive disease | 192 | 2014-2018 | 8th | 6 | 19A(6) | PEN-COT-TET |
| Pakistan | Invasive and non-invasive disease (113), carriage(75) unknown (2) | 190 | 2010-2020 | 5th | 10 | 7B(1), 14(1), 10A(2), 19A(2), 19F(4) | PEN-COT-ERY-TET |
| Peru | Invasive disease (188) and Carriage (419) | 607 | 2006-2011 | 17th | 10 | 19A(8), 24F(2) | PEN-COT-ERY-TET |
| PNG | Invasive disease | 191 | 1989-2017 | Not found | - | - | - |
| Poland | Invasive disease | 189 | 2007-2013 | 10th | 5 | 19A(3), 19F(1), 23A(1) | PEN-COT-ERY-TET |
| Russia | Invasive and non-invasive disease (80), carriage(99) | 179 | 2011-2018 | 17th | 3 | 19F(3) | PEN-COT-TET |

| | | | | | | | |
|---|---|---|---|---|---|---|---|
| South Africa | Invasive disease (2919) and Carriage (1695) | 4614 | 2005-2014 | 9th | 14 8 | 3(6), 10A(1), 14(137), 19A(1), 23A(2), 24(1) | PEN-COT-ERY-TET |
| The Gambia | Invasive disease (398) and Carriage (1249) | 1647 | 1993, 1996-2014 | Rare | 3 | 3(2), 10A(1) | PEN-COT-TET |

Non-PCV13 serotypes were underlined.

Table S8. The relationship between pneumococcal 24F driver lineages and antibiotic consumption.

| | Major driver of serotype 24F | | Narrow and broad spectrum penicillin | | Macrolide[a] | |
|---|---|---|---|---|---|---|
| Countries | GPSC (CC) | Resistance profile | DDD[b] per 1000 | Ratio | DDD per 1000 | Ratio |
| Denmark | 6 (CC156) | COT | 4,781 | Ref | 664 | Ref |
| Argentina | 10 (CC230) | PEN-COT-ERY-TET | 4328 | 0.9 | 770 | 1.2 |
| France | 10 (CC230) | PEN-COT-ERY-TET | 8,522 | 1.8 | 1,311 | 2.0 |
| Japan | 106(CC2572) | ERY | 634 | 0.1 | 1,525 | 2.3 |
| Lebanon | 10 (CC230) | PEN-COT-ERY-TET | 6544 | 1.4 | 765 | 1.2 |
| Spain | 10 (CC230) | PEN-COT-ERY-TET | 10,389 | 2.2 | 998 | 1.5 |

Erythromycin belongs to the macrolide class of antibiotics. COT, cotrimoxazole; PEN, penicillin; ERY, erythromycin; TET, tetracycline

[a]Macrolide is a class of antibiotics which includes erythromycin

[b]DDD, defined daily dose. The DDD estimates in 2015 are extracted from https://resistancemap.cddep.org/AntibioticUse.php
